# AI-aided holographic flow cytometry for label-free identification of ovarian cancer cells in the presence of unbalanced datasets

**DOI:** 10.1063/5.0153413

**Published:** 2023-06-07

**Authors:** F. Borrelli, J. Behal, A. Cohen, L. Miccio, P. Memmolo, I. Kurelac, A. Capozzoli, C. Curcio, A. Liseno, V. Bianco, N. T. Shaked, P. Ferraro

**Affiliations:** 1 Dipartimento di Ingegneria Elettrica e delle Tecnologie dell'Informazione (DIETI), Università di Napoli Federico II, 80125 Napoli, Italy; 2 Institute of Applied Sciences and Intelligent Systems “E. Caianiello,” CNR-ISASI, Via Campi Flegrei 34, 80078 Pozzuoli, Napoli, Italy; 3 Department of Chemical, Materials and Production Engineering of the University of Naples Federico II, Piazzale Tecchio 80, Napoli 80125, Italy; 4 Tel Aviv University, Ramat Aviv, 6997801 Tel Aviv, Israel; 5 Unit of Medical Genetics, Department of Medical and Surgical Sciences (DIMEC), University of Bologna, Via Massarenti 9, Bologna 40138, Italy; 6 Study and Research Center on Gynecological Neoplasias, Department of Medical and Surgical Sciences (DIMEC), University of Bologna, Via Massarenti 9, Bologna 40138, Italy; 7 Center for Applied Biomedical Research (CRBA), University of Bologna, Bologna 40138, Italy

## Abstract

Liquid biopsy is a valuable emerging alternative to tissue biopsy with great potential in the noninvasive early diagnostics of cancer. Liquid biopsy based on single cell analysis can be a powerful approach to identify circulating tumor cells (CTCs) in the bloodstream and could provide new opportunities to be implemented in routine screening programs. Since CTCs are very rare, the accurate classification based on high-throughput and highly informative microscopy methods should minimize the false negative rates. Here, we show that holographic flow cytometry is a valuable instrument to obtain quantitative phase-contrast maps as input data for artificial intelligence (AI)-based classifiers. We tackle the problem of discriminating between A2780 ovarian cancer cells and THP1 monocyte cells based on the phase-contrast images obtained in flow cytometry mode. We compare conventional machine learning analysis and deep learning architectures in the non-ideal case of having a dataset with unbalanced populations for the AI training step. The results show the capacity of AI-aided holographic flow cytometry to discriminate between the two cell lines and highlight the important role played by the phase-contrast signature of the cells to guarantee accurate classification.

## I. INTRODUCTION

Performing an accurate early detection is one of the main challenges in modern medicine when dealing with cancer related diseases. Today, medicine mainly relies on the standard tissue biopsy and histological examination to diagnose the suspect mass. However, tissue biopsy is a highly invasive and slow diagnostic tool, which cannot be frequently repeated on the same patient, and might fail to reveal tumor heterogeneity. In this framework, the paradigm of liquid biopsy (LB) is becoming crucial due to the unique advantages it offers, since it is a noninvasive, fast diagnostic tool based on the search of the tumor derived material in a peripheral blood sample. For example, the detection of circulating tumor cells (CTCs), i.e., cells detached from the primary tumor or from metastasis that enter the bloodstream, not only allows to locate and classify the disease potentially at an earlier stage as compared to standard diagnostic imaging methods but it allows also to obtain precious information to guide the patients' management. Most common techniques used to identify and classify CTCs, as reported by Refs. 1–10, are based on the recognition of a known, specific indicator that univocally distinguishes them from the healthy cells composing the bloodstream. Commonly used indicators are cancer-specific surface antigens; however, given the high tumoral heterogeneity, antigen-based methods often lead to false negative results. To overcome biased epitope analyses, methods based on the recognition of peculiar functional CTC properties are being developed, such as matrigel invasion capacity^11^ or high glycolytic metabolism,^12^ which to date, do not offer the high-throughput levels required for screening programs. On the other hand, using next-generation sequencing (NGS) analysis to identify tumor-specific mutations in the bloodstream offers greater potential in terms of throughput, but often depends on cumbersome CTC enrichment steps and requires cell destruction. All the above-mentioned strategies, even if commercially available and objective of extensive research efforts, have the big disadvantage of requiring *a priori* information about the CTC, that might not be available, making the search highly specific for a particular type of cancer, considering the great heterogeneity of tumors. In addition, they often do not allow for high-throughput screening tests, which is of primary interest in clinics. The latter is mainly due to the fact that a CTC pre-enrichment step is required to detect rare CTCs in the bloodstream, typically occurring with a frequency of 1–10 cells per 1 ml.^13^ Finally, most of these approaches imply CTC destruction, not allowing viable cell recovery after the analysis. Therefore, single cell analysis approaches based on flow cytometry (FC) have garnered a huge interest for the potentiality they offer to overcome these issues.^14,15^

FC is a widely used technique for examining, characterizing, and sorting cells that are let flow suspended in a fluid and analyzed one-by-one. Therefore, FC is one of the most appropriate ways of investigating biological materials that naturally live in suspension, e.g., all blood cells. The standard parameters measured over the cells are obtained by exploiting scattering processes or the optical readout in transmission microscopy modes; imaging-FC (IFC) allows to obtain much more useful information about the morphology and possibly the inner structures of each cell. In fact, IFC allows to combine the high-throughput imaging capabilities of conventional flow cytometry with the specificity of the single-cell analysis. All this information available is precious in tasks like detection and classification of CTCs and allows to exploit high content analysis such as deep learning (DL) approaches or conventional machine learning (ML); accordingly, in this work, we will focus on image-based LB. Since biological specimens such as CTCs are transparent and, therefore, not suitable for brightfield microscopy imaging, the standard way used to investigate these samples requires the use of labels to mark them, typically linked to fluorescence emission. This method, even though highly specific, has some important drawbacks. First, staining procedures must be set up to label the cells; this requires highly trained personnel, increases the costs of the analysis for supplying and for properly disposing reagents, and slows down the imaging process. Second, the labels alter the physiological behavior of the specimens and may create phototoxicity; although this impairment does not exclude the possibility of *in vivo* imaging, it may mine the reliability of the detection process itself, changing the way in which the CTCs respond to a designated detection marker. Moreover, fluorescence-based markers create photobleaching. These important drawbacks caused a shift in attention to label-free ways to perform imaging of transparent samples, and the most promising class of methods in this framework is referred to as quantitative phase imaging (QPI).^16,17^ QPI methods are particularly interesting because they yield a morphometric characterization of the samples; the produced images, the so-called quantitative phase-contrast maps (QPMs), are measurements of the optical path delays produced by the specimens. This gives access to a wide variety of measured features relative to single cells such as the optical thickness, biovolume, and dry mass distributions, which might be of primary interest for detection and classification and that would be unavailable with merely qualitative methods. Moreover, since the phase-shift information encodes 3D volumetric information into a single image, the refractive index 3D distribution can be retrieved if different QPMs relative to different projections are acquired and tomographic reconstructions are performed.^18,19^ Among the wide plethora of QPI approaches, such as phase-shifting interferometry, Fourier ptychography, transmission intensity equation (TIE), spatial light interference microscopy (SLIM), or gradient light interference microscopy (GLIM), digital holographic microscopy (DHM) is one of the most affirmed techniques. As the entire information of the complex wavefront is recorded, DHM is independent of the focus at which the image is acquired. Therefore, all the cells visible in the field of view can be dealt with as they show up, with no need of real-time focusing, as the best focus will be adjusted via numerical post processing. This peculiarity makes DHM the most suitable candidate to image cells through the continuous stream of microfluidic systems.

**FIG. 1.**
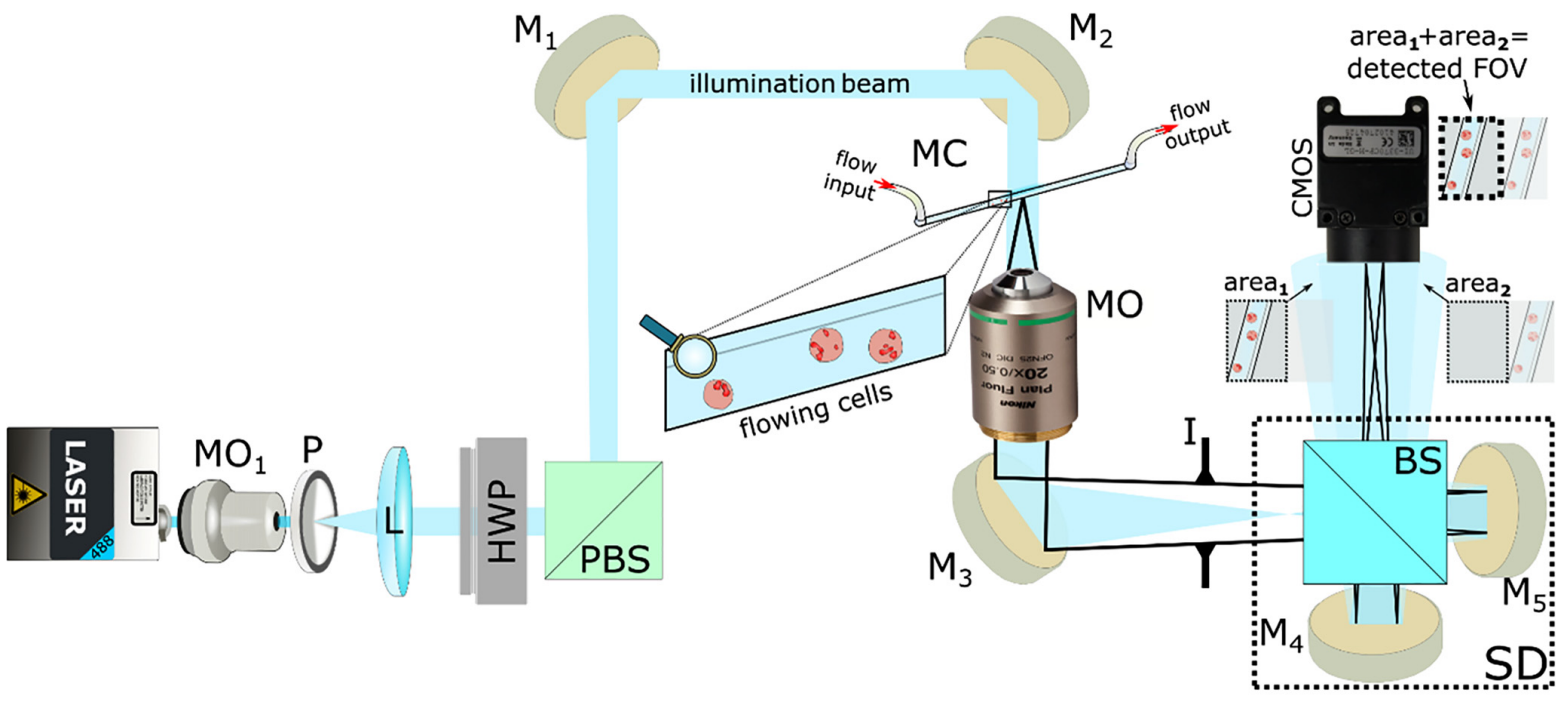
Sketch of experimental arrangement. MO1—focusing microscope objective; P—pinhole; L— collimating lens; HWP—half-wave plate; PBS—polarizing beam splitter; MO—microscope objective; MC—microfluidic chip; Ms—mirrors; I—iris diaphragm; BS—beam splitter; CMOS—camera; SD—shearing device. The image in front of the MO provides a zoom into the MC with cells flowing inside the channel. Inserts in front of the CMOS illustrate replicas arising from the SD. The highlighted image portions (area1 and area 2) represent overlapping areas detected by the CMOS, which provide correct holographic signature.

In flow, DHM is the one that better combines the need to obtain very fast, high-throughput morphometric measurements with high accuracy and noninvasiveness. The last decade has seen the growth and widespread use of lab-on-a-chip (LoC) technology, which allows a very accurate flow engineering, manipulation of small amounts of liquid samples, from microliters to nanoliters, and enables establishing laminar flows along predetermined pathways. Hence, DHM in optofluidic configurations well suits the discussed need to capture and classify rare tumoral cells in the bloodstream. In this work, different classification techniques will be analyzed and compared, namely, a ML approach and a DL approach, both applied to classify QPM reconstructions of cells in continuous flow inside a LoC, using the setup sketched in Fig. 1. In particular, artificial intelligence (AI) is used to perform classification between model cell lines, i.e., ovarian cancer (OC) cells (A2780) and monocytes (THP-1), whose AI training sets were unbalanced in ration 1 monocyte every 6.36 OC cell. It is important to note that it is simple to filter out red blood cells, whereas it is harder to discriminate CTCs from monocytes because of their similarity in size; therefore, the discrimination problem is non-trivial. Both the conventional ML and the DL classification approaches are shown to provide excellent accuracies, higher than 90% in tests, with a slight but non-neglectable advantage of the DL approach. Moreover, we carry out a feature engineering study, and we point out how the features extracted from the quantitative phase-contrast distribution are among the most informative available.

## II. DATASET

We employed a DH setup in transmission microscopy mode to acquire sequences of model cell lines flowing inside a microfluidic circuit. The holographic flow cytometer is implemented as a Michaelson interferometer, as sketched in Fig. 1 and described in detail in Sec. VI. Acquired holograms were processed in order to obtain the sample complex amplitude in sharp focus for each cell. This process involves hologram demodulation, backpropagation and automatic refocusing, phase extraction and compensation of optical aberrations, phase unwrapping, and denoising. Details on the hologram processing pipeline are provided in Sec. VI. The phase-contrast images obtained in this way were the input of the ML and DL classifiers. In the end, for the analysis, a total of 2951 QPMs was considered, composed of 2550 A2780 model cancer cells and 401 THP-1 monocytes. The dataset was relatively small, and the populations were not balanced. One of the objectives of the analysis is to assess whether it is possible to classify data with poor datasets and to assess if deep learning methods are more suitable for this task than machine learning ones. The dataset is split between a training set (97.3%) and a test set (2.7%) through a holdout partition.

## III. MACHINE LEARNING APPROACH

### A. Feature analysis

In the framework of a ML approach, all the QPMs available were numerically processed to extract features useful for the classification task. Alongside with the morphological features (area, herein expressed in pixels, eccentricity, perimeter, expressed in pixels) and texture-based features (homogeneity, energy, kurtosis, skewness, and entropy), which are commonly considered for classification, a new group of features was added, which are available thanks to the quantitative nature of the DHM readout. In particular, we measured the maximum, minimum, mean value, and standard deviation of the values measured over the QPMs.

**FIG. 2.**
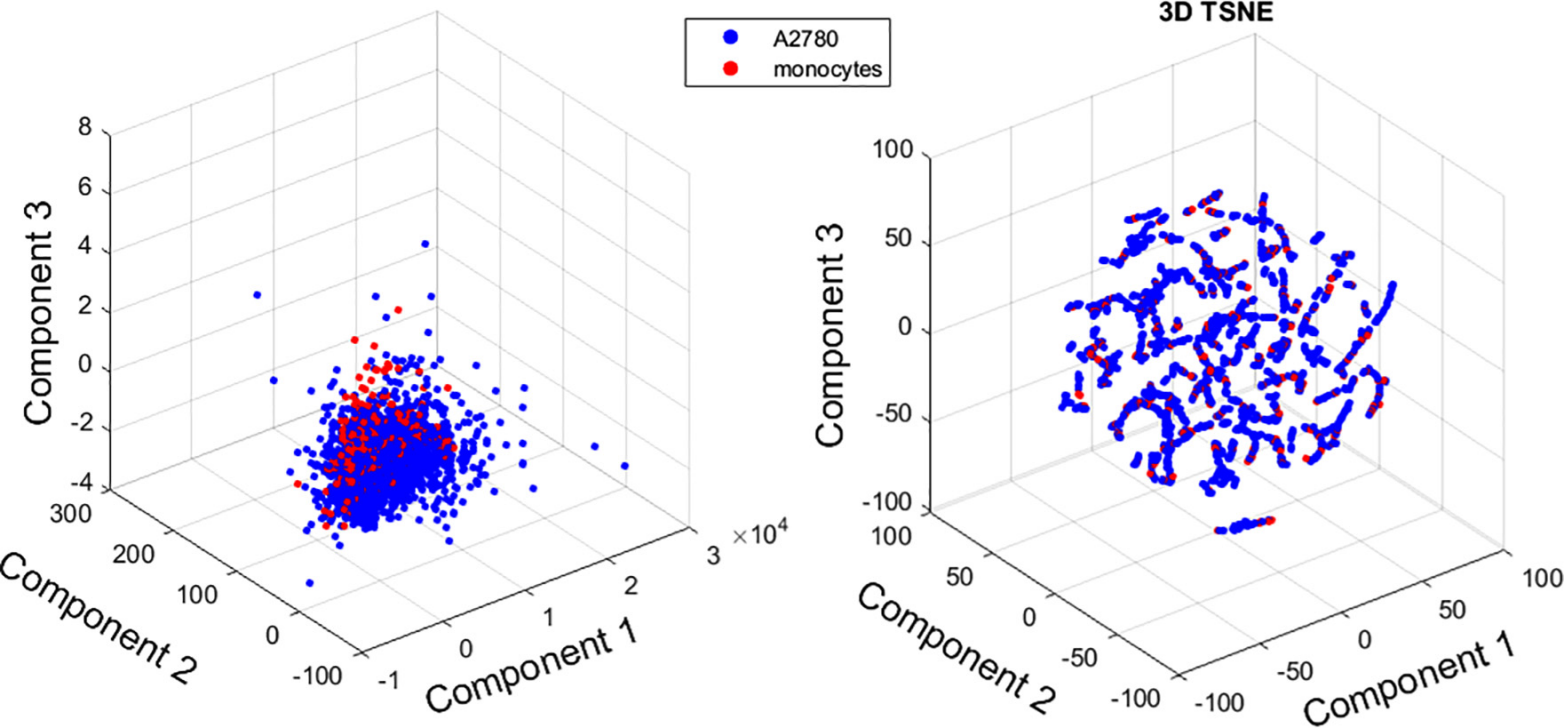
PCA and T-SNE carried out over the data before feature selection to assess the complexity of the classification task by observing the natural data clustering.

The morphological features were extracted starting from a binarized version of the QPMs. The texture features were extracted starting from the gray level co-occurrence matrix (GLCM), which expresses how combinations of discretized values (gray levels) of neighboring pixels are distributed along one of the image directions. Finally, the quantitative parameters associated with the sample optical thickness were directly measured over QPMs. It is worth pointing out again that these features are intrinsically linked to the holographic imaging paradigm, as they are measurements made over the samples of physical quantities that are not accessible using conventional imaging approaches, which would only provide qualitative information. In total, 12 features were considered for the classification. A first investigation was carried out to understand if the considered data tended to naturally cluster into the two species or not. To do that, both a PCA and a T-SNE^20^ analysis were performed to reduce the dimensionality of the feature space and to visually inspect the data. Figure 2 reports the results of this analysis and, clearly, the cells under investigation do not tend to naturally cluster. This preliminary test suggests that the classification problem tackled here is not trivial.

**FIG. 3.**
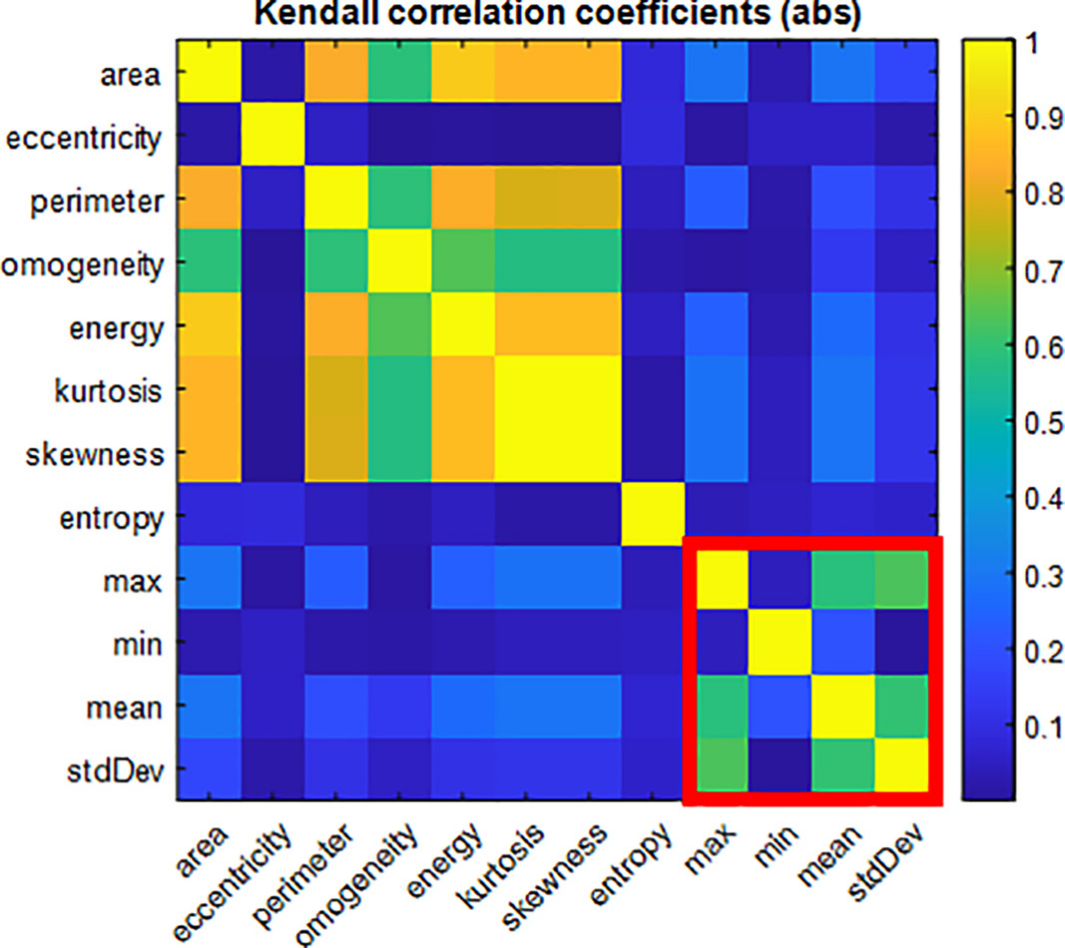
Absolute value of Kendall correlation coefficient among the inspected features. The red square marks out the QPI features.

**FIG. 4.**
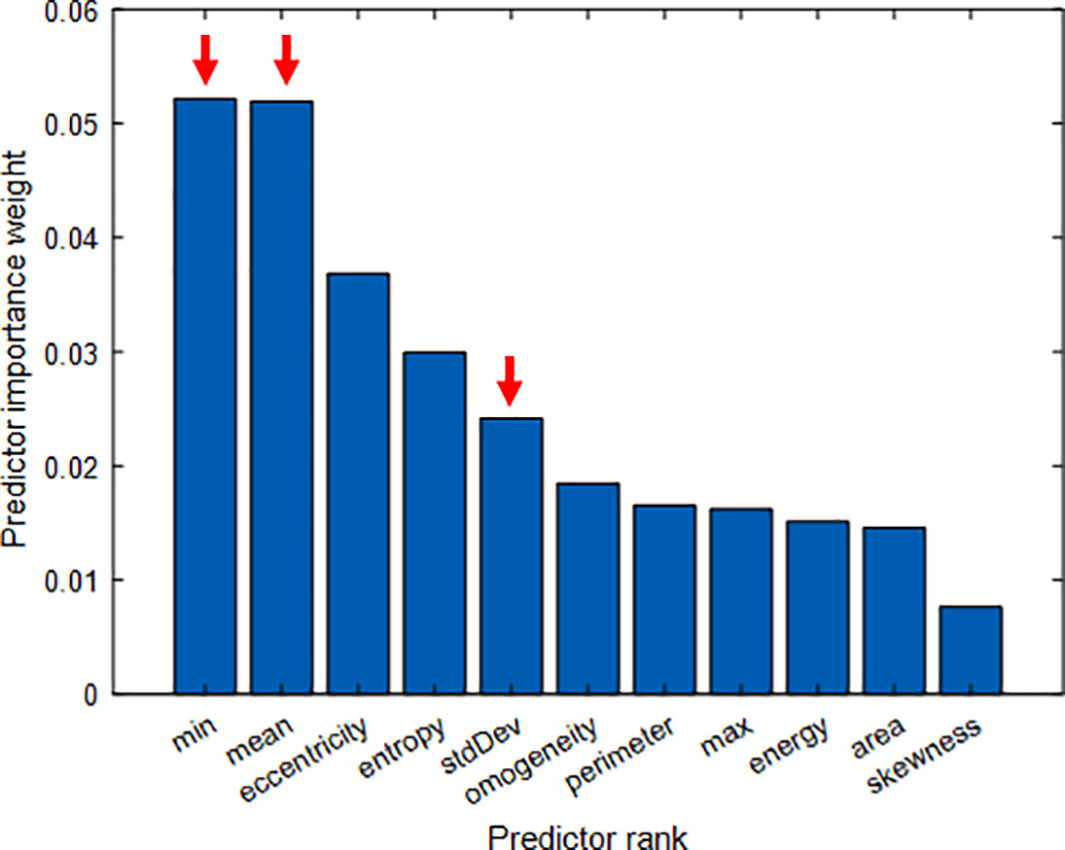
Results of Relief analysis performed over the features to assess their significance. The red arrows point out some of the QPI features, which are among the most informative.

**FIG. 5.**
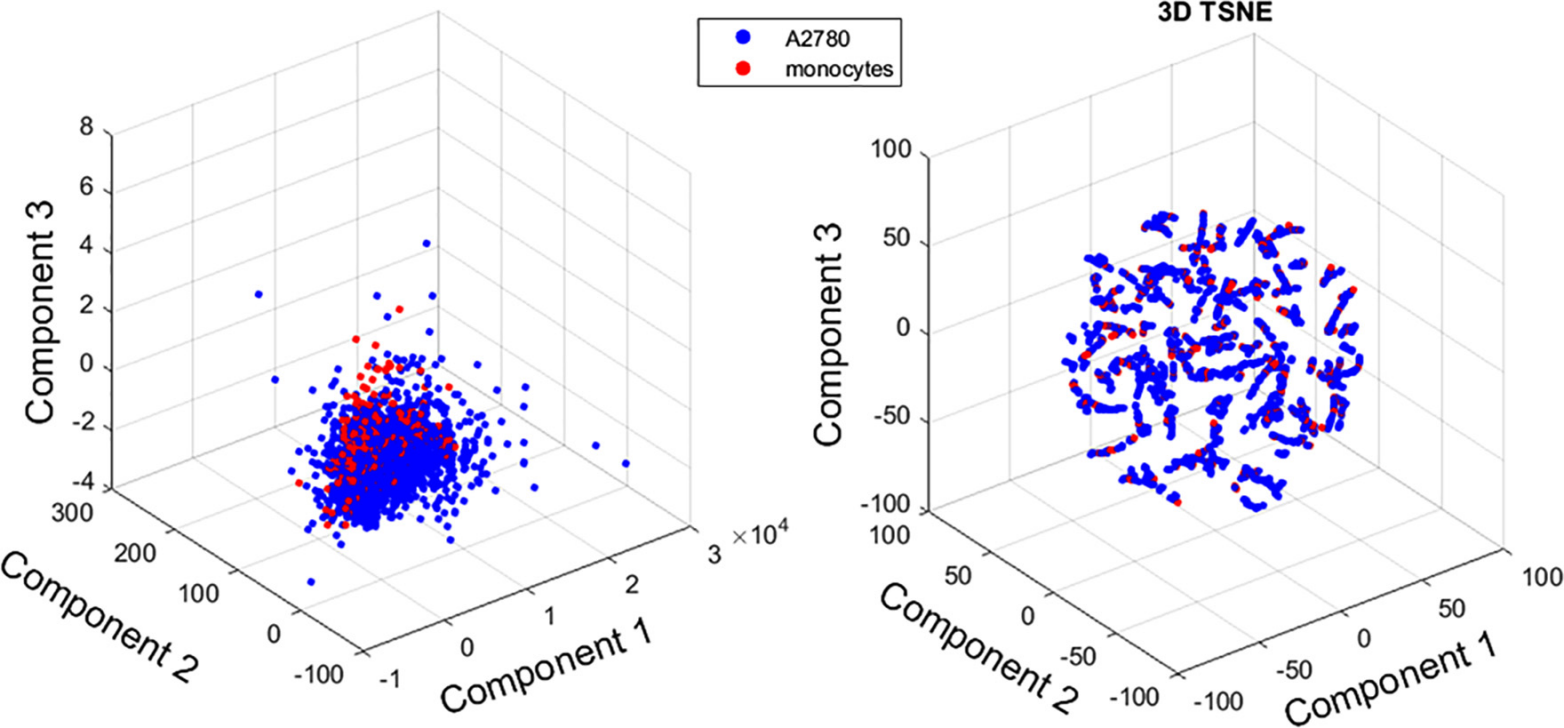
Results of PCA and T-SNE analysis after the feature selection process.

Consequently, an analysis over the extracted features was performed to understand their level of significance, following the approach proposed by Valentino *et al.*^21^ First, the Kendall correlation coefficient was evaluated over all the features. The choice of using Kendall correlation was due to the non-normal distribution of the data and their low numerosity. Figure 3 shows the absolute value of the evaluated correlation coefficient. Some considerations are now in order. Observing the matrix, it can be seen that morphological and texture features do show some correlation with statistical relevance (p-value < 0.05). Conversely, the QPI features (highlighted by the red square) show a low value of intracorrelation and, in particular, a very low value of correlation with the other features; both these observations are of statistical relevance (p-value < 0.05). From a mere classification standpoint, this means that useful information is being carried out by the QPI features and that all of them are, in principle, worth further analysis. Considering only the values of correlation coefficients with statistical relevance (p-value < 0.05), a threshold R = 0.9 was set to discriminate between correlated and uncorrelated features. The features that had a correlation coefficient greater than the threshold were only the kurtosis and the skewness. As a consequence, the kurtosis feature was discarded since it was judged not enough informative in the ensemble. Subsequently, we used the Relief Algorithm^22^ to quantitatively understand which were the most informative features among the ones under examination. Figure 4 reports the result of this ranking where the red arrows point out that, as expected, some of the QPIs features are among the most informative. Finally, according to the pipeline presented in Refs. 21 and 23, we again performed PCA and T-SNE over the data to assess whether an improvement was made over the clustering of the data via the feature selection process. As it can be appreciated from Fig. 5, no improvement is obtained in terms of clustering, coherent with the observation that all the features are more or less significant except for the discarded redundant one.

### B. ML classification results

**FIG. 6.**
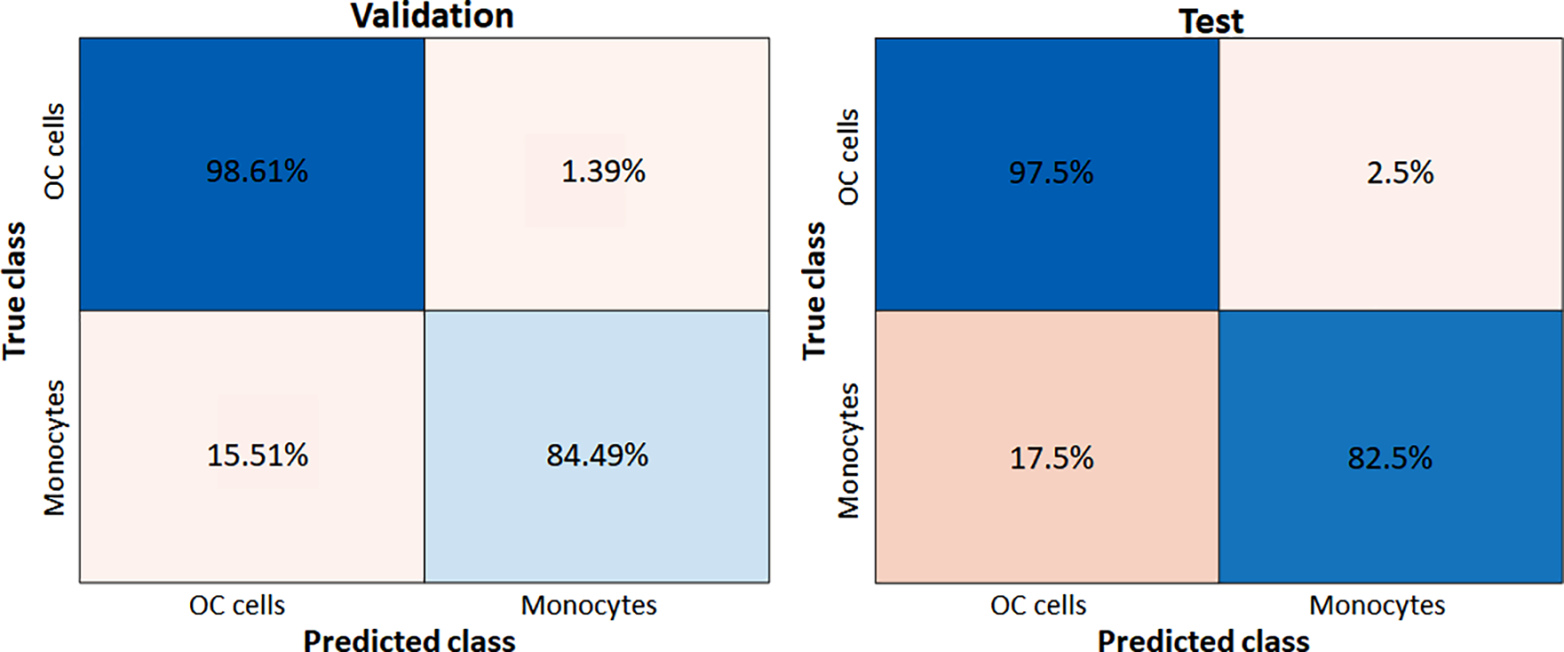
Validation and test confusion matrices relative to the Cubic SVM classifier when all the features are employed.

The ML classification task between the monocytes and the model CTCs was conducted via the Classification Learner App released by MathWorks. In this analysis, different conventional models are trained and evaluated for the classification. The training is performed over the training set discussed above with a cross validation made via a 10-class k-folding. Table I reports the validation and test accuracy for the considered classifiers in different scenarios. In particular, we intended to investigate the relevance of the different group of features for the classification; therefore, we selectively excluded a certain class of features among the three available (morphological, texture, QPIs) while considering the other two and repeated the training/test procedure to compare the results. First, all the features were used for the classification task. Then, the sole morphological features were excluded; afterward, the texture features were excluded and the morphological reintegrated, and finally, all the features were included except for the QPIs. As expected, the best validation accuracy is obtained when all the features are employed, coherent with the results of the feature ranking procedure. However, removing the QPIs features results in a consistent drop in both the validation and test accuracy, confirming that these features are the most discriminative, which justifies in full the use of a QPI method for coping with this classification issue. In general, the values of accuracy are very high with a significative decrease in the test accuracy, symptom that some overfitting over an insufficient dataset might be present for a machine learning classification task. Figure 6 reports the validation and test confusion matrix relative to the Cubic SVM classifier, which in general, shows the best performance (Table I), for the case in which all the features are employed for the classification, which we already assessed to be the best case. As indicated by the test confusion matrix, there is a high number of monocytes incorrectly classified as OC cells (17.5%); conversely, just 2.5% of the model CTCs is incorrectly classified as monocytes and, therefore, not identified. However, it is worth pointing out that given the high rarity of CTCs in the bloodstream compared to monocytes, such a high percentage of misclassification might lead to a complete loss of the CTCs. Accordingly, given the relevance of the false negatives as compared to false positives in a cancer-diagnostic application such as the one of interest, in the evaluation of different classification approaches, the priority was set in finding a classificator providing the minimum amount of false negative results rather than of false positives. Concluding, the non-optimal classification results obtained via ML are coherent with the almost absent natural clustering of the data, symptom that a ML approach might not be the most effective to handle the problem complexity.

**TABLE I.** Validation and test accuracy percentage evaluated for different classifiers considering different feature groups.

	All features		No morphological		No texture		No QPI	
Classificator	Validation	Test	Validation	Test	Validation	Test	Validation	Test
Fine tree	93.3	85.0	93.9	83.8	90.8	81.2	89.3	75.0
Medium tree	93.8	81.2	93.7	80.0	92.2	73.8	90.3	67.5
Coarse tree	92.2	72.5	92.2	72.5	90.1	63.7	90.8	62.5
Linear discriminant	93.3	77.5	92.4	75.0	92.5	76.2	90.5	67.5
Quadratic Discriminant	94.7	88.8	93.2	78.8	93.5	82.5	90.5	76.2
Linear SVM	93.9	80.0	93.8	80.0	92.6	78.8	91.4	66.2
Quadratic SVM	96.7	88.8	96.6	87.5	94.8	87.5	92.3	72.5
Cubic SVM	96.8	90.0	96.1	87.5	95.3	85.0	93.3	80.0

## IV. DEEP LEARNING APPROACH

### A. Networks' architecture

To perform the DL classification task, we have chosen to resort to convolutional neural networks (CNNs) pre-trained on ImageNet,^25^ since the dataset was relatively small. Pre-training has made the classification task easier to perform. The performance of the networks was compared for five different net architectures: MobileNet V2,^24^ MobileNet V3 small, MobileNet V3 large,^26^ and ResNet-18.^27^ Some CNNs may suffer from low diagnostic performance due to vanishing-gradient and divergent-gradient problems, which obstruct information transmission from shallow layers to deep layers of the network. ResNet solves this problem by identifying shortcut connections, skipping certain layers while providing great generalization performance with a relatively small number of parameters. Indeed, ResNet has been successfully used for many medical image classification tasks.^28^

**FIG. 7.**
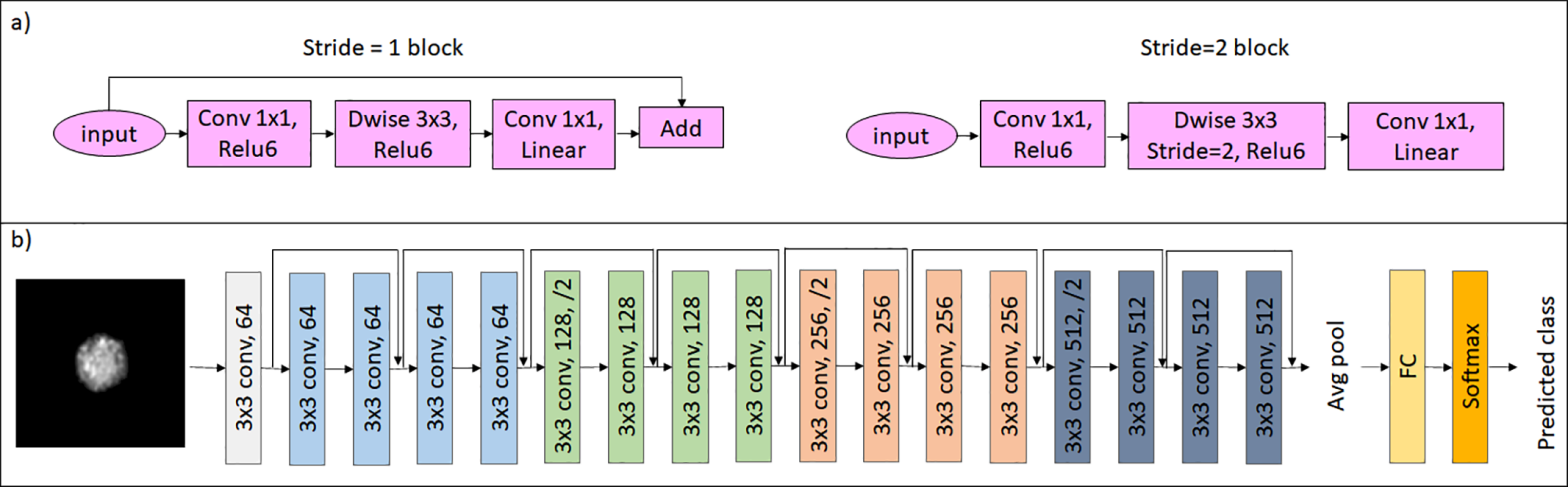
Figures showing the (a) details of the MobileNet V2 architecture and (b) ResNet-18 architecture used.

The architecture of the original ResNet-18 is shown in Fig. 7(b). There are a total of 18 layers in the network (17 convolutional layers, a fully connected (FC) layer, and an additional SoftMax layer to perform the classification task). The convolutional layers use 3 × 3 filters, and the network is designed in such a way that if the output feature map is the same size, then the layers have the same number of filters.

The basic block of MobileNet is the inverted residual bottleneck. The MobileNet V2 inverted residual bottleneck block, shown in Fig. 7(a), consists of a 1 × 1 point-wise convolution expanding the input to a higher dimensional space, followed by a 3 × 3 depth-wise convolution. Finally, a second point-wise convolution reduces to a smaller dimension and residually connects to the input layer. The outputs of all stages passes through a ReLu6 activation function.

The MobileNet V3 inverted residual bottleneck is similar to V2 with the addition of the squeeze-and-excite block,^29^ which executes average pooling to a vector composed of two FC layers and multiplies the produced vector with the original matrix. Furthermore, in MobileNet V3, the ReLu6 activation is replaced with the hard-swish activation function. MobileNet V3 small and MobileNet V3 large differ by the amount of inverted residual blocks (11 in small vs 15 in large) and of number of channels in each block. Our input to the net are the QPMs without any further processing, and the last FC layer uses a SoftMax activation function to produce the classification score prediction y∈[0,1].

### B. Implementation details

The training process was performed over the training set with a fivefold cross validation. For each fold, the network was trained for 10 epochs with a batch size of 16, using the ADAM optimizer,^30^ the Cross Entropy (CE) loss, and a learning rate of $\lambda =10^{-4}$.

### C. DL classification results

**FIG. 8.**
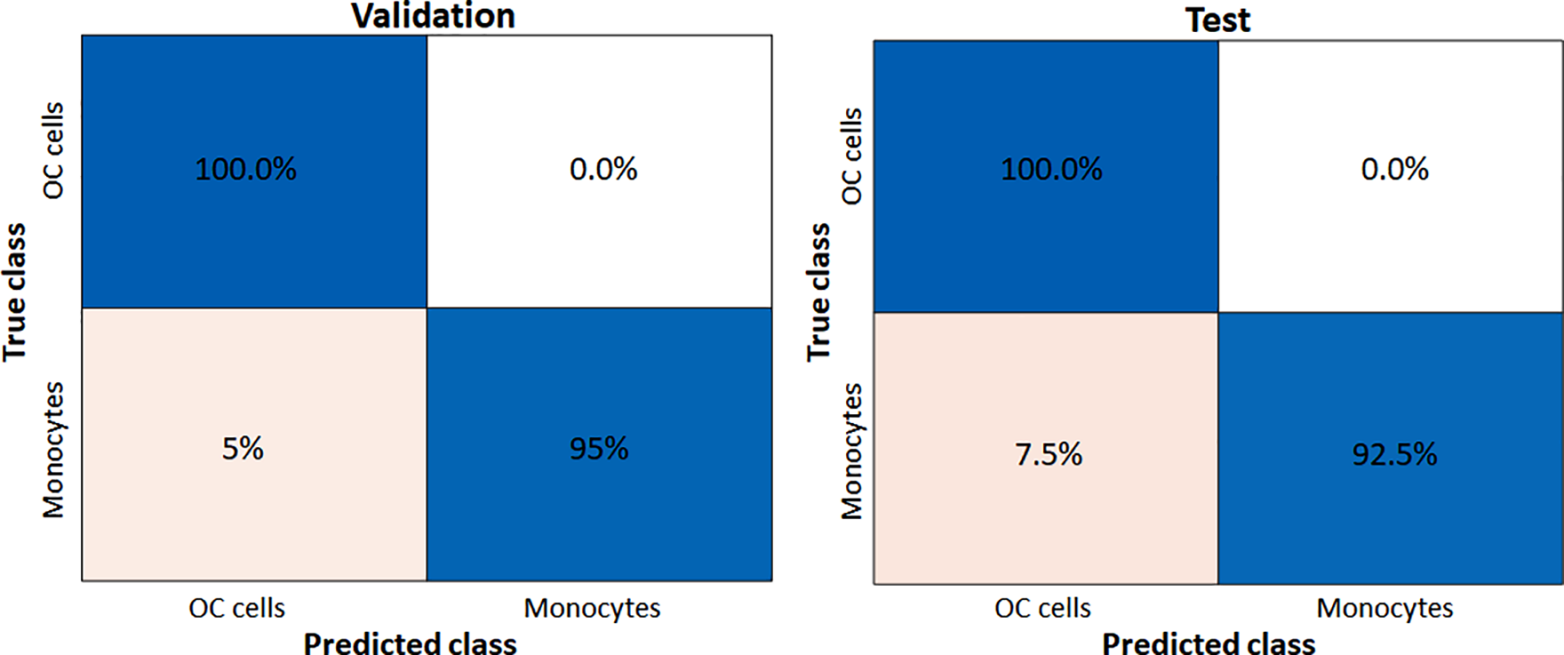
Validation and test confusion matrices relative to MobileNet-V2 classifier.

Table II reports the validation and test accuracy over all folds for each network architecture. In general, the values of accuracy are high, specifically in comparison to the conventional ML approach. The networks have similar results, but we can see a slight improvement in the accuracy when using ResNet-18 and MobileNet V2 as compared to other networks. Figure 8 reports the validation and test confusion matrix relative to MobileNet-V2 classifier. In both validation and test confusion matrices, there is an elevated number of monocytes incorrectly classified as OC cells (5% and 7.5%, respectively), while there were no model OC cells unidentified. This agrees with the fact that the population of the cells were not balanced.

**TABLE II.** Results summary of the validation and test accuracy over all folds.

Network architecture	Validation accuracy	Test accuracy
MobileNet V2	95.2	93.2
MobileNet V3 Small	79.2	79.7
MobileNet V3 Large	88.6	87.9
ResNet-18	94.0	94.0

## V. CONCLUSIONS

In this work, the problem of discrimination between cancer and blood cells in the case of unbalanced datasets was discussed. This is a crucial issue for LB, a new diagnostic tool that recently arose as a powerful alternative to tissue biopsy. We proposed a system implementing a holographic flow cytometer aided by AI for the analysis of reconstructed data as paradigm to perform LB. In particular, we studied the problem of discerning between A2780 OC cells and monocytes, i.e., blood cells of comparable sizes that are difficult to separate by existing microfluidic sorting devices. First, we carried out an investigation on the available features. Studying the capability to cluster the two populations, we found a non-negligible overlap between them in the features' space, i.e., the problem of discrimination is not trivial and, therefore, learning approaches must be used. We compared two discrimination approaches, one based on conventional ML and one on DL. Although both methods provided good values of accuracy for classification, greater than 90%, the DL method demonstrated superior results. It is worth pointing out that conventional ML strongly benefits from the holographic features added to the conventional morphological and textural parameters. We discussed the role played by the unique features that we could extract by the phase-contrast map. The improvement of the DL method over the ML method, although apparently marginal in terms of percentage accuracy increase, is particularly important, given the rarity of CTCs in the bloodstream and the diagnostic relevance of a correct classification. In other words, in the framework of LB, it is pivotal to minimize the rate of false negatives in the presence of rare events to be detected (i.e., the occurrence of a CTC). In this sense, we judge the improvement brought by DL as non-negligible. Considering both the ML and DL classification results, the higher rate of monocytes incorrectly classified as OC cells might be explained by the unbalanced dataset. However, given the aforementioned need to maximize in deployment, the number of CTCs correctly classified, training the classifiers with an elevated number of OC cells makes the procedure robust in terms of capability of recognizing them in a real LB scenario, where a false negative result should be avoided since it might concur to a late diagnosis. Results have shown that AI-aided holographic flow cytometry is a promising solution for LB of OC. Future works will go in the direction of increasing the complexity of the sample, e.g., we will benchmark the system in the case of spiked blood samples and, finally, in the case of patient-derived LB.

## VI. METHODS

### A. Experimental setup description

The experimental setup is a lateral-shearing digital holographic microscope based on the Michelson geometry. Figure 1 shows a simplified sketch of the laboratory setup, where the linearly polarized laser beam (sapphire SF, λ=488 nm; spectral linewidth < 1.5 MHz => coherence length ≈100 m) is spatially filtered by a microscope objective MO1 (Newport 20×/0.40) and a pinhole P (diameter 10 μ m) and collimated by a positive lens L (focal distance 125 mm). A half-wave plate (HWP) and a polarizing beam splitter (PBS) allow fine light-power adjustments. The beam is further reflected by mirrors (M), and it subsequently illuminates the sample inside the microfluidic chip (MC, Straight 4-channel Mini-Luer Chip (P/N 10000091); channel dimensions = 200 μm wide × 200 μm deep × 58.5 mm; lid thickness = 140 μm). An automatic syringe-pump system (CETONI Syringe Pump neMESYS 290N) has been implemented to achieve a laminar flow. Here, a glass syringe containing the sample is connected by the plastic inlet tubing to the MC, followed by the plastic outlet tube and the waste glass. The flow rate during measurements is set in the range of 5−20 nl/s.

The sample flowing inside the MC is subsequently imaged by a microscope objective (MO; Nikon, 20×/0.50, Plan Fluor) directly into the plane of the camera's chip (CMOS; UI-3370CP-M-GL, 2048 × 2048, 5.5 μm square pixels). Here, the shearing-device (SD), providing the lateral shear,^31^ consists of a beam splitter (BS) and two mirrors M4 and M5; thus, two duplicate images of the studied sample are created. The first image arises from the optical path BSM4BS and the second arises following the path BSM5BS. These two replicas are directed toward the camera with slightly different inclination angles and lateral displacements due to the different tilts of the mirrors M2 and M3. Consequently, a portion of the observed field (Fig. 1; area1) serves as a signal beam, while the sample-free area in the replica (Fig. 1; area2) serves as a reference beam. If the coherence conditions of the interfering waves are satisfied, the interference fringes arise, thus enabling single-shot off-axis holographic recordings and subsequent numerical reconstructions. In the present experimental configuration, the lateral shift among both replicated images is comparable to the dimensions of the used CMOS chip, so only one of the replicas is observed in a snapshot image. However, in principle, it is feasible to exploit both replicas to enhance the quality of the retrieved complex amplitude of the studied object, as was proposed elsewhere.^32^ The Iris diaphragm (I), inserted between the MO and CMOS, reduces the amount of unwanted back-reflections and stray light. The lateral magnification of the imaging system, measured by a positive USAF 1951 amplitude line target, was established as 55, and the expected theoretical lateral resolution in the object space can be approximated as 0.82λ/NA≈0.8 μm.

### B. Hologram processing

**FIG. 9.**
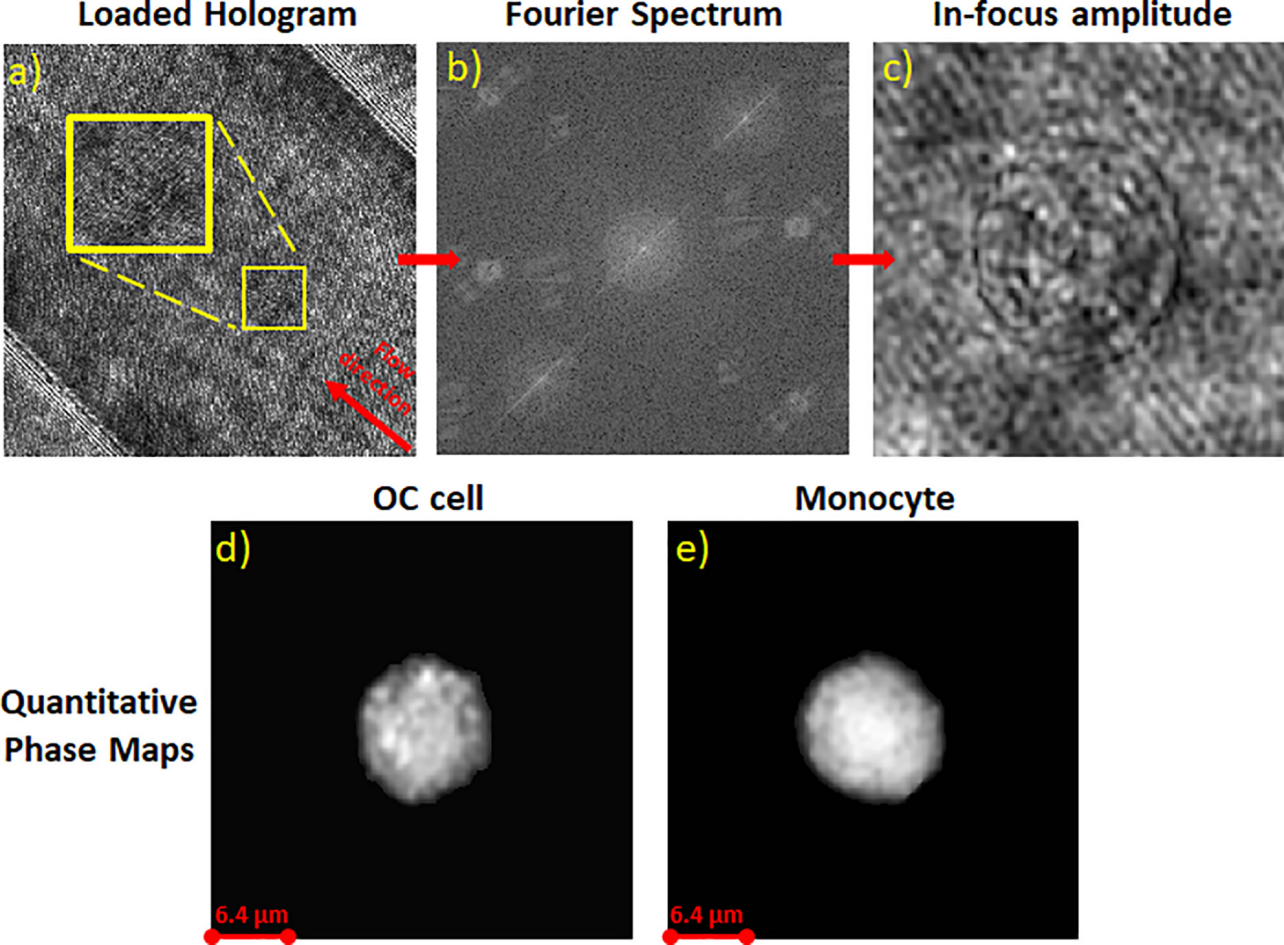
Outline of the reconstruction pipeline: (a) acquired hologram (b), Fourier spectrum of the holograms with the three orders visible (c), in-focus amplitude of the complex field obtained after the refocusing procedure, and (d) and (e) QPMs of an OC cell and a monocyte.

Imaging tests were carried out using monocyte cells (THP-1) and OC cells (A2780). The samples were prepared as described in Ref. 33. Cell suspensions were allowed to flow inside the channel, and holographic video sequences were acquired separately for each cell type. Once the acquisition was accomplished, the holograms were numerically processed to obtain the QPMs. The processing is made via a pipeline extensively described,^18^ and here reassumed and sketched in Fig. 9. First, the holograms were apodized and, subsequently, the valuable diffraction order was extracted from the spectrum [Figs. 9(a) and 9(b)]. The retrieved complex amplitude was numerically refocused making use of the angular spectrum approach^34^ employing the Tamura coefficient as metric^18^ [Fig. 9(c)]. The aberrations introduced by the optical system were corrected using a reference hologram, e. g., a cell-free hologram, which was processed with the same steps as the object hologram up to the refocusing stage and then subtracted in phase from the object hologram, obtaining the wrapped 2π-modulo phase map. For every cell flowing into the field of view, a variable number of frames was considered, from one to four depending on the availability. A square area of size 384 pixels was selected, centered around the centroid of the cell in the considered frame. Afterward, the phase maps were unwrapped using the PUMA algorithm,^35^ denoised^36^ to attenuate the speckle correlated noise and binary masked to isolate the cell profile over the background. The images obtained in this way were the input of the ML and DL classifiers [Figs. 9(d) and 9(e)]. A total of 2951 QPMs, composed of 2550 A2780 cancer cells and 401 THP-1 monocytes, was considered for the analysis. The dataset was relatively small, and the populations were not balanced. One of the objectives of the analysis is to assess whether it is possible to classify data with poor datasets and to assess if DL methods are more suitable for this task than ML ones. The dataset is split between a training set (97.3%) and a test set (2.7%) through a holdout partition.

## Data Availability

The data that support the findings of this study are available from the corresponding author upon reasonable request.
